# Supplementary material for: Are carbon emissions trading and green financial instruments synergistic? -Comprehensive quantitative research based on content analysis
Source: PLoS One. 2024 Mar 7;19(3):e0298601. doi: 10.1371/journal.pone.0298601 (PMC10919601; doi:10.1371/journal.pone.0298601)
Supplement: S1 Data — (ZIP) [file pone.0298601.s001.zip › Data/地级市Z_F工作报告.zip!/║╙▒▒/╩»╝╥╫»╩╨/└┤╫╘╣1⁄2╓┌║┼í╛┬φ┐╦╩2╛▌═°í┐_2012.docx]

各位代表：

现在，我代表市人民政府向大会作政府工作报告，请予审议，并请各位政协委员和列席会议的同志提出意见。

一、2011年工作回顾

刚刚过去的一年,是实施“十二五”规划开局之年，是中共石家庄市第九次代表大会胜利召开之年。面对国内外错综复杂的形势，我们在省委、省政府和中共石家庄市委的坚强领导下，坚持以科学发展为主题，以加快转变经济发展方式为主线，紧紧围绕转型升级、跨越赶超两大任务，全力以赴保增长、调结构、控物价、惠民生，较好地完成了市十二届人大四次会议确定的目标任务,实现了“十二五”良好开局。

综合实力跃上新台阶。预计全市生产总值完成4000亿元,增长12%;全部财政收入完成488.9亿元，其中地方一般预算收入完成221亿元，分别增长26%和35%;规模以上工业增加值完成1700亿元，实现利税770亿元，分别增长16%和25%；社会消费品零售总额完成1640亿元，增长18%。全民创业成效明显，民营经济持续健康发展。中东西三大区域协调发展，中部区域隆起效应逐步显现，东部区域整体突破取得较大进展，西部山区发展加快步入良性循环。

重点建设取得新成效。全市固定资产投资完成3000亿元，增长25%以上。重点项目建设取得新进展，71个项目开工建设，73个续建项目超额完成年度投资计划，格力电器、神威现代中药制剂等52个项目竣工投产。正定新区、东部产业新城、空港工业园、大西柏坡基础设施和产业项目建设同步推进，取得实质性进展。五大产业基地和南部工业区建设步伐加快，华药白蛋白、青岛啤酒等49个项目竣工投产。“20+X”重点商贸项目和“10+X”物流项目加快推进，综合保税区申办取得阶段性进展。省会夜经济成效显著，一批美食休闲广场、特色商业街建成开业。西柏坡高速公路一、二期竣工通车，西阜高速公路开工建设，石家庄国际机场航站楼改扩建工程主体完工。

转型升级迈出新步伐。传统产业升级改造步伐加快，完成技改投资680亿元，增长25%。战略新兴产业实现增加值267亿元，增长25%。产业园区进档升级提速，石家庄·中国半导体光谷建设全面启动，7个经济开发区和11个产业集聚区列入省级序列，产业聚集效应日趋明显。高新区二次创业成效显著，示范带动作用明显增强。现代物流、总部经济、服务外包、文化旅游等现代服务业规模壮大，服务业增加值占GDP总量的比重达到40%以上。科技创新能力明显提高，新增高新技术企业35家，市级以上企业（工程）技术中心36个，取得省以上科技成果205项。淘汰落后产能任务超额完成,节能减排目标如期实现，大气和水环境质量持续改善，全年市区二级以上天气达到320天，市域5条主要河流水质基本达到省定标准。

城镇面貌发生新变化。围绕城市建设上水平，实施重点工程80项，完成投资1500亿元。城市规划引领作用增强，聘请国内外一流规划大师和设计团队，编制完成了21项重要规划和单体设计。城市综合交通体系日臻完善，铁路新客站及六线隧道主体工程基本完工，中华北大街、胜利大街等11条道路改造全面完成。城市居住条件明显改善,103个保障性安居工程项目、45048套保障性住房开工建设。城中村拆迁改造稳步推进，637栋城中村回迁楼开工建设，305栋竣工、2.3万户居民回迁新居。15个旧住宅小区环境综合整治全面完成。良村热电、鹿华热电等7个项目全部竣工，新增供热能力3710万平方米。城市标志性景观打造成效明显，勒泰中心、河北航空城等十大城市综合体建设加快推进，列入全市风貌特色十大工程的园博园、会展中心、体育中心开工建设，以万达广场建成开业为标志，城市风貌特色增添新亮点。城市生态环境明显改观，全面推进“一河两环”工程，环城水系全线通水，滹沱河市区段16公里的河道具备蓄水条件，全长11.6公里的滨河景观大道竣工通车，民心河二期南栗明渠建成投用，新建公园、广场8座，新增绿地708公顷，建成区绿地率达到41.5%。城市精细化管理效果显著，以管理重心下移为重点的大城管体制基本理顺，万米网格化管理深入实施，主城夜景亮化提档升级。城区主干道全部实现机械化清扫，108条路段实现全天候保洁。县城扩容提质加快推进，736个项目开工建设，完成投资478亿元，县城人居环境、承载功能和聚集能力进一步提升。全市城镇化率达到51.5%。

统筹城乡取得新进展。认真落实各项强农惠农政策，各级财政用于“三农”支出达到68.6亿元，增长37.2%。农业综合生产能力稳步提高，粮食亩产、总产分别达到472.7公斤和545.9万吨，粮食连续八年丰收，单产总产双超历史最高水平。畜牧、蔬菜、果品三大优势产业规模不断扩大，质量品质明显提高。农业产业化经营总量达到800亿元以上，增长12%。大力实施太行山和环省会绿化工程，森林覆盖率达到30%。农田水利建设加力提速，完成10座水库除险加固，新打维修机井3586眼，发展节水灌溉30万亩，改造中低产田5.9万亩。农村生活环境进一步改善，新改建农村公路917公里，新开通市区至组团县（市）公交线路10条。新民居建设稳步推进，新增省级新民居示范村250个，建成了一批设施配套、美观宜居、群众满意的新民居。

改革开放实现新突破。国企改革向纵深推进，金刚集团、宝石集团等一批大中型企业成功重组，5家集体企业和18个城中村集体组织完成改制。行政审批制度改革不断深化，全市321项行政许可和非行政许可审批事项削减到245项。医药卫生体制改革深入推进，全市乡镇卫生院综合改革基本完成，农村普遍实行大病统筹，全市44个政府办社区卫生服务机构和所有乡镇卫生院全部实行了基本药物制度。投融资体制改革取得新进展，渤海银行在省会设立分行,新增小额贷款公司11家，石药集团等5家企业成功发行债券，以岭药业等3家企业挂牌上市。对内对外开放不断扩大，石洽会、正博会、药博会等大型经贸活动成功举办，格力电器、可口可乐等一批国际国内产业巨头前来我市投资办厂。全市实际利用外资8亿美元，引进市外资金500亿元；外贸进出口总额完成130亿美元，增长18%。

民生幸福实现新提升。围绕实施八项重点民生工程，市财政用于民生支出295.8亿元，增长38.8%。城镇居民人均可支配收入达到20120元，农民人均纯收入达到7560元，分别增长10%和15%。城镇新增就业10.5万人，下岗失业人员再就业3.75万人，转移农村劳动力15.2万人，应届高校毕业生就业4.13万人，城镇登记失业率控制在3.79%以内。养老、医疗、失业等五项保险覆盖面进一步扩大，新型农村养老保险和城镇居民养老保险分别在11个县(市)试点，失业保险市级统筹开始实施，新农合参合率达到96.8%，所有农村实施了门诊统筹并做到出院即报。城镇退休职工养老保险、新农合筹资、城乡低保标准继续提高。建立保障标准与物价上涨挂钩联动机制，困难群体生活得到保障。全市新增廉租住房补贴家庭2000户，分配入住廉租房5034套。解决了40万农村人口的饮水安全问题，3.5万农村贫困人口实现稳定脱贫。为民承诺的27件实事基本完成。我市居民幸福指数在全国294个城市中排名第一，人民群众对省会的归属感、认同感、自豪感大大增强。

社会事业取得新进步。教育事业加快发展，学前三年教育入园率达到88.3%，义务教育均衡发展，高中教育优质资源进一步扩大，职业教育水平不断提升，高等教育质量稳步提高。484所中小学校舍安全工程竣工投用，幼儿师范学校顺利实现升格转型，职教园区建设加紧推进。山区教育扶贫工程成效明显，28所寄宿制学校建成投用，1.8万余名贫困山区学生实现了集中就读。推出一批文化精品，戏剧《女人九香》、动画电影《西柏坡》、纪录片《石破天惊》等优秀作品荣获国家级大奖。第六届国际动漫博览交易会成功举办，动漫实训基地与考试中心开始运行，动漫产业产值达到12亿元。新建农家书屋1636个，市图书馆、博物馆、美术馆等公益场馆免费开放，“彩色周末”、“石演大舞台”等群众文化活动丰富多彩。全市乡镇卫生院全部配备了基本的诊断设备和救护车辆。4041所农村标准化卫生室建成投用，基本实现“一村一室”的目标。深入开展“首席医师”进社区、千名医师下乡村活动，“十大”名医、名护、名科等评选命名深受市民称赞。全民健身运动广泛开展，竞技体育取得优异成绩。深入实施计划生育惠民行动，低生育水平保持稳定。国防动员和双拥共建深入开展，“六城同创”统筹推进，精神文明建设成果丰硕，获全国文明城市提名资格。妇女儿童、老龄慈善、扶贫助残等事业不断发展，民族宗教、外事侨务、防震减灾、气象人防、社科档案等各项事业都取得新成绩。

政府建设得到新加强。依法行政水平不断提升，自觉接受人大法律监督和政协民主监督。办理人大代表建议367件、政协委员提案629件，办结率均达100%。深入开展创先争优活动，强力推进效能革命，发展环境进一步优化。大力推行行政权力公开透明运行，专项资金、小金库、公车治理工作取得明显成效。认真落实市县乡三级干部大接访制度，一批信访积案和遗留问题得到解决。食品药品监管专项治理成效明显。重特大安全生产事故得到遏制。社会管理创新综合试点工作成效显著，社会治安防控体系进一步完善，110座综合警务服务站建成投用，全市政治稳定、社会安定、人民安居乐业。

各位代表！去年以来，我市经济社会发展取得的优异成绩，是省委、省政府和市委正确领导的结果，是全市人民团结奋斗、共同努力的结果。在此，我代表市政府，向全市人民，向人大代表、政协委员，向各民主党派、工商联、无党派人士、人民团体和社会各界人士，向驻石人民解放军、武警官兵，向中直、省直机关驻石各单位，向所有关心石家庄发展的国内外朋友，致以崇高的敬意和衷心的感谢！

在充分肯定成绩的同时，我们必须清醒地看到，我市经济社会发展还存在一些突出的困难和问题，主要是：区域经济社会发展还不够协调，部分企业经营困难，经济增长下行压力加大，市场物价高位运行，城乡居民生活受到影响，资金紧张矛盾突出，土地瓶颈制约趋紧，外贸出口形势严峻，节能减排任务艰巨。与此同时，制约我市发展的产业结构档次低、发展方式粗放、自主创新能力不强、社会事业发展滞后等深层次矛盾更加凸显，县域经济实力不够强，城乡差距还比较明显。新型城镇化和城市现代化水平还比较低，省会城市基础设施和功能尚须下大力完善，城市管理的规范化、标准化、精细化水平还比较低。改善民生和社会建设方面还有不少亟待解决的问题。政府职能转变还不够到位，优化发展环境任重道远。对此，我们一定要高度重视，采取有力措施，切实加以解决。

二、2012年工作的总体要求和主要目标

今年是全面落实市第九次党代会精神的第一年，是深入实施“十二五”规划承上启下的关键一年，做好今年的政府工作意义十分重大。

综合分析今年的形势，既有许多有利条件，也面临严峻挑战。世界经济在曲折中复苏，新一轮科技革命孕育重大突破，国际范围内产业结构调整和转移进一步加快，有利于我们在更大范围、更宽领域、更高层次上参与经济合作与竞争。国家继续实施积极的财政政策和稳健的货币政策，着力扩大内需，优化融资结构，为我们争资金、上项目提供了难得机遇和空间。京津冀区域经济一体化纳入国家“十二五”规划，冀中南地区被列为国家层面的重点开发区域，省委、省政府对省会建设高度重视，省第十一届人代会第五次会议明确提出“提升省会城市功能，打造河北第一窗口”，这些都为我们全面提高城市规划、建设和管理水平，加速建设现代化省会城市、京津冀区域中心城市和生态宜居城市，提供了前所未有的历史性机遇。特别是市第九次党代会描绘了我市未来发展的宏伟蓝图，开启了建设幸福石家庄的新征程。全市广大干部群众推动科学发展、创造幸福生活的认识更加统一，转型升级、跨越赶超的信心更加坚定，聚精会神搞建设、一心一意谋发展的氛围更加浓厚。同时也必须看到，国际国内形势依然复杂严峻，世界经济增速减缓、美国经济持续低迷、欧洲主权债务危机不断发酵，经济复苏的不确定性因素增多。我国经济增长下行压力和物价上涨压力并行，经济持续向好的基础尚不稳固。加之我市经济结构性矛盾与实体经济实力不足相互叠加，短期问题与长期矛盾相互交织，我们面临着“转型”和“赶超”的双重压力。对此，我们必须切实做到头脑更加清醒、谋划更加周密、准备更加充分，在挑战和压力面前抢抓机遇，在转型升级、攻坚克难之中谋求突破，努力在新一轮竞争中抢占先机、争取主动、跨越赶超，奋力实现更好更快地发展。

今年政府工作的总体要求是：坚持以邓小平理论和“三个代表”重要思想为指导，深入贯彻科学发展观，全面落实中央经济工作会议和省第八次党代会精神，围绕市第九次党代会作出的战略部署，以建设幸福石家庄为目标，锁定转型升级、跨越赶超两大任务，深入实施中东西三大区域经济协调发展战略，加快工业化、城镇化和农业现代化进程，着力稳增长、控物价、调结构、抓创新、惠民生、促和谐，推动全市经济社会更好更快发展，努力在建设经济强省、和谐河北中发挥带头作用，以优异成绩迎接党的十八大胜利召开。

今年全市经济社会发展的主要预期目标是：地区生产总值增长11%，全部财政收入增长14%，其中一般预算收入增长15%；全社会固定资产投资增长19%，社会消费品零售总额增长18%，外贸进出口总值增长10%，实际利用外资增长10%；单位生产总值能耗和二氧化碳排放量均下降3.89%，化学需氧量、氨氮和二氧化硫排放总量分别削减1%、1%和0.5%；城镇居民人均可支配收入增长11%，农民人均纯收入增长11%；城镇登记失业率控制在4.5%以内；居民消费价格涨幅控制在4%左右；人口自然增长率控制在7.9％。以内。

实现今年经济社会发展目标，必须突出把握好“稳中求进”的工作总基调，在稳定增长中加快转型，在转型升级中跨越发展。坚持把转型发展作为加快推进产业升级的主攻方向，更加注重调整经济结构和转变发展方式；坚持把创新发展作为实现跨越赶超的中心环节，更加注重体制机制创新和科技创新；坚持把绿色发展作为建设“两型”社会的根本途径，更加注重节能减排和环境保护；坚持把统筹发展作为推动经济社会发展的基本方略，更加注重推进城乡一体化和中东西区域协调发展；坚持把和谐发展作为打造全省首善之区的必然要求，更加注重保障和改善民生。

三、2012年主要工作任务

今年全市改革发展任务十分繁重，必须全面落实国家宏观调控政策，多措并举保增长，积极作为促转型，攻坚克难求跨越，努力开创省会建设的新局面，着力做好以下几个方面的工作：

（一）深入实施中东西发展战略，全力构筑区域经济协调发展新格局。坚持顺应自然规律，遵循经济规律，发挥比较优势，优化产业空间布局。中部区域，重点发展高新技术产业、新兴产业和现代服务业，以打造东部产业新城为重点，着力整合“两区三基地”（高新区、藁城开发区，循环化工、装备制造、纺织服装基地）优势资源，凝聚发展合力。市区大力发展现代物流、电子商务、研发设计、服务外包、金融保险等生产性服务业，提升商贸流通、总部经济、社区服务、文化娱乐、休闲保健、餐饮住宿等生活性服务业。东部区域，以大上工业为主攻方向，以产业聚集区建设和招商引资为突破口，坚持改造提升传统产业与发展壮大新兴产业并重，依据国家产业政策，强化集群集约，延长产业链，提升价值链，努力在推进产业集群、打造各具特色的支柱产业上实现新的突破。西部区域，以发展绿色工业、生态农业、农副产品加工业和休闲旅游业为重点，突出生态屏障建设，加快富民强县步伐，在改造提升传统加工业、做大做强旅游业和消除区域贫困上取得实质性进展。

（二）加强重点项目建设，提升经济发展的支撑力。牢固树立抓项目就是抓发展的理念，坚持政策聚焦、要素聚集、力量聚合，高起点、高标准推进项目建设。今年全市安排省市重点项目160项，总投资2900多亿元，年度计划投资600亿元以上。加快续建项目建设进度，在保质量、保安全的前提下，科学调度，全力推进，确保格力三期、以岭药业中药产业化等35个项目竣工投产；对已具备开工条件的重点项目，进一步优化资源要素配置，破解瓶颈制约，给予重点保障，争取石炼化工业气体岛一期、南车集团石家庄产业园、LED系列应用产品产业化等70个重点项目尽快开工建设；谋划一批具有支柱和引领作用的立市、立县大项目。瞄准国家产业政策和投资方向，切实加强项目储备管理，确保进入储备库项目达到1000项以上。认真抓好项目包装论证工作，加大与国家、省重点项目计划的对接力度，力争更多的项目进入国家和省计划盘子。

（三）做大做强工业经济，增强经济发展的拉动力。按照“二产抓提升”的要求，进一步强化工业主导地位，加快工业强市步伐。实施传统产业升级计划。以精深加工、节能降耗、重组整合为着力点，继续开展行业企业对标行动，对照国内外行业先进标准，加强研发力量，提升配套能力，延长产业链条，加大技改力度，年内重点实施千万元以上技改项目200项以上。实施新兴产业倍增计划。以核心关键技术研发、自主技术产业化为着力点，启动实施信息产业升级、生物产业创新发展、半导体照明产业化、新材料特色产业链壮大四大工程，加快发展物联网，培育壮大战略性新兴产业。突出抓好生物医药、光电信息产业，力争将生物医药产业培育成我市工业经济的第一支柱产业，将光电信息产业打造成中国北方最大的光电信息产业集群，全市战略性新兴产业增加值增长25%以上，力争用三年时间实现战略性新兴产业规模倍增。实施“千亿园区”培育计划。以国家级高新区、省级工业聚集区和经济开发区为载体，重点抓好石炼化千万吨炼油综合改造、安瑞科一期、石煤机扩能等一批重点项目，省级工业聚集区、经济开发区主营业务收入增长20%以上。力争经过3年努力，高新技术开发区、藁城经济开发区、循环化工基地均达到千亿级园区规模。实施“百亿企业”塑造计划。以工业50强为依托，认真落实工业扶持政策，鼓励引导骨干企业实施规模扩张，推动生产要素向优势企业和产品集中，着力培育一批国际国内知名品牌，加速打造一批具有核心竞争力的大型工业“航母”，主营业务收入百亿元以上企业达到7家。力争经过3年努力，华药、石药等4家企业销售收入超500亿元，全市“百亿级企业”达到14家。实施节能减排攻坚计划。坚持结构调整、技术进步、管理创新三措并举，铁腕治污、刚性降耗，确保完成节能减排约束性指标。突出抓好新老“双三十”、“双千”企业的治理，加强对重点耗能企业的能源审计，强化对环保重点监控企业的在线监控，推进污染减排重点工程建设。严格控制高能耗和产能过剩企业扩大产能，禁止新上高排放项目，加快淘汰落后产能。

（四）毫不放松地抓好“三农”工作，力促农业增效、农民增收。按照“一产抓特色”的要求，扎实推进农业现代化，促进农业增效、农民增收、农村发展。提升粮食生产能力。认真落实强农惠农政策，大力开展高产创建活动，加快500万亩粮食生产核心区建设。今年全市粮食总产保持在505万吨以上，力争实现“吨粮市”目标。提高农业综合效益。以优质、高产、高效、生态、安全为目标，发展壮大畜牧、蔬菜、果品三大优势产业，在农产品出“特色”上创品牌、增效益。加大农业产业化投入力度，支持180家市级农业产业化龙头企业做大做强，重点抓好中粮可口可乐、天天乳业等一批投资亿元以上产业化项目。进一步完善基层农技服务网络，加强农产品质量安全体系建设。改善农村生产生活条件。年内完成治理河流4条，除险加固病险水库15座，整治河道堤防20公里，治理水土流失160平方公里，发展节水灌溉30万亩，解决40万农村人口饮水安全问题。加快农村垃圾污水处理设施建设，完成新一轮农村电网改造，促进农村无线通讯全面升级。提高农民收入水平。大力实施帮扶农民增收工程，吸引农民进城务工经商，有效扩大农民非农就业。加强农民就业培训，积极发展劳务经济，增加农民工资性收入。鼓励支持种养能手、返乡农民工创办实业，增加经营性收入。认真落实国家新10年扶贫开发纲要，全面启动260个扶贫开发重点村整村推进计划，年内确保3.5万人实现稳定脱贫。加快统筹城乡发展步伐。以省会周边、新区、县城及产业基地等区域为重点，大力推进城乡规划、产业发展、基础设施、公共服务、劳动就业和社会保障、社会管理“六个一体化”，逐步实现公共资源均衡配置，生产要素自由流动，经济社会协调发展。积极稳妥地推进新民居建设，建成一批高标准农村新型社区。

（五）加快发展现代服务业，培育壮大服务型经济。按照“三产抓拓展”的要求，力促服务业发展提速、比重提高、结构提升。加快发展现代物流业。突出抓好南部综合物流聚集区、正定商贸物流产业聚集区建设，重点实施“10+X”现代物流项目，确保晋冀北方物流交易中心等一批项目竣工投用，冀中现代物流配送中心等一批项目开工建设，加速打造全国重要物流节点城市。大力发展金融保险业。积极吸引国内外大型商业银行在省会设立分支机构，争取县级农村信用社改农村商业银行在我市先行试点，发展小额贷款公司、村镇银行等金融机构，积极搭建银企合作平台，努力扩大信贷投放，创新灵活多样的金融产品，支持培育一批企业上市融资。做大做强旅游业。全面规划整合旅游资源，打造精品线路，完善配套设施，加大宣传推介力度，着力提升西柏坡、苍岩山、嶂石岩、抱犊寨、伏羲台、封龙山、正定古城等十大旅游精品景区管理水平，重点推进驼梁—五岳寨—天桂山5A级景区创建，推出一批旅游特色村、星级农家院，组建一批大型旅游集团，努力把石家庄建设成为国内外热点旅游目的地城市。培育壮大服务外包和会展业。高标准建设石家庄国际服务外包开发区，力争把服务外包业打造成为全市新的经济增长点。大力繁荣会展经济，打造石洽会、皮博会、动博会、药博会、正博会等知名会展品牌，会展经济总量增长50%以上。改造提升传统商贸服务业。加快大型商贸综合体和特色商业街建设，突出抓好“20+X”重点商贸流通项目，年内万象天成、希尔顿等五星级酒店落成开业。加强夜经济精品项目建设，全面提高夜经济的档次和品位，进一步提升省会的繁华度。坚持多措并举，有效扩大城乡消费需求。落实国家房地产调控政策，促进房地产市场健康发展。

（六）推进城镇建设上水平，倾力打造一流省会城市。围绕打造河北第一窗口，完善配套设施，提升城市功能，聚集优势产业，强化精细管理，全力推进城镇建设上水平、出品位、生财富。主城提升增亮点。加快新源财富大厦、勒泰中心、中国美食街等一批集高端商务办公、大型商业购物、休闲娱乐于一体的大型城市综合体和中央商务区建设。加快新客站广场拆迁及周边路网建设和商业开发。加快胜利大街地下商业街建设进度。继续实施城中村拆迁改造工程，推进回迁楼建设。新区建设出形象。按照“低碳、生态、智慧”的理念，加快正定新区建设，大力推进会展中心、体育中心等26个功能性项目建设。进一步加大路网、公建配套、景观整治力度，年内初步形成两环、三横、三纵的主路网框架，力争河北出版集团、华侨大厦等总部经济项目“五一”前开工，尽快使新区建设有一个大的变化。完善设施强功能。加快西阜高速公路建设，开工建设平赞、石太二通道和南绕城高速公路。推进干线公路升级改造，加快三环辅道建设，确保石铜公路建成通车，启动正无公路藁城、无极段和京港澳高速市区连接线建设。进一步完善交通配套设施，启动空港综合交通枢纽、新火车站综合客运站建设。扩大热源热网覆盖面，加快旧小区供热管网改造，新增供热能力2350万平方米。园林绿化添新景。改造提升文化广场等5座公园，启动环城水系东线、西线景观绿化、游船码头工程。提速民心河二期建设，确保年底前9.55公里河道整治全面竣工。在城市主干道沿线和重要节点见缝插绿多植树，新建10处游园绿地，新增城市绿廊绿道20公里，中心城区人均绿地面积达到5.8平方米以上。城市管理上水平。积极推进城市管理体制机制创新，加快实现综合执法，构建管理与执法高效统一的大城管格局，大力提升城市管理的标准化、规范化、精细化水平。引入市场化运作模式，以竞争机制助推市政公用服务质量提升。增加环卫作业机械设备，把二环路纳入机械化保洁范围，努力提高主次干道全天候保洁率。提升裕华路、中山路、中华大街等重要路段和繁华商业区域的夜景景观。加大对建筑渣土乱撒乱倒和建筑工地扬尘噪音的治理力度，努力营造洁净舒畅、清爽宜人的城市环境。围绕县城扩容升级，着力推进县城重大功能性项目建设。同时，着力打造一批工业型、商贸型、旅游型特色小城镇，加快形成具有石家庄特点、体现时代特征的现代城镇体系。

（七）加大改革开放和创新力度，增添科学发展的动力和活力。继续深化重点领域和关键环节改革，拓展对外开放的广度和深度，推动经济发展向创新驱动转变。加快改革攻坚步伐。大力推进国有企业改革重组，支持骨干企业兼并扩张。完善公司法人治理结构，盘活国有资产，实现保值增值。切实抓好投融资平台建设，加强政府债务管理。加大综合保税区跑办力度，力争尽快得到国家批准。全面推进事业单位分类改革，完成事业单位分类清理规范工作。全方位扩大对外开放。进一步加大招商引资力度，深化与京津及长三角、珠三角发达地区的合作，发挥外侨作用，搭建合作交流平台，密切与国际友城和港澳台的联系，引进一批大公司、大集团，提高利用外资的规模和水平。积极开拓国际市场，优化出口商品结构，加快出口基地建设，促进出口稳步增长。提高科技创新水平。以创建国家创新型城市为目标，加大产业技术创新力度，培育更多的自主知识产权、自主品牌和创新型企业，重点支持高新区创建国家创新特色园区，新认定高新技术企业20家，新增市级以上创新型企业30家，新培育市级以上工程技术研究中心10家。深入实施“十大人才工程”，面向国内外引进一批科技领军人才和创新创业团队，立足本市重点培养一批企业经营管理人才、技能型人才、农村实用人才，真正让人才优势成为石家庄的第一竞争力。发展壮大民营经济。认真落实促进民营经济发展的各项政策措施，进一步完善服务体系，加快融资担保、人才培训、科技信息、创业辅导基地建设。继续实施中小企业成长工程，对重点民营企业和成长型中小企业，在土地、资金、技术上给予重点支持，打造一批“小巨人”企业。大力推进全民创业，积极扶持小微企业发展，努力营造能人创企业、百姓创家业、干部创事业的浓厚氛围。

（八）大力发展社会事业，全面加强和谐石家庄建设。坚持以人为本，优化公共资源配置，推进公共服务均等化，促进社会和谐进步。提高教育发展水平。实施学前教育三年行动计划，年内新改扩建农村规范化公办幼儿园100所。继续推行义务教育学校标准化建设，改造提升200所中小学校园环境，实现县域义务教育初步均衡。突出抓好学生营养餐改善，加强中小学校车安全管理。巩固提高高中阶段教育普及水平，加快第15中学迁建工程建设，打造一批社会公认、河北一流的优质高中。推进职业教育规模化、集团化办学，启动建设正定新区职教园区。加强高等院校特色学科、重点学科建设，全面提高教育质量。加快发展文化事业。深化文化体制改革，推动文化大发展大繁荣。继续开展道德模范、中国好人和感动省城人物等评选活动。以推进“六城同创”为载体，深入开展创建全国文明城市活动。加大投入力度，加强公共文化基础设施建设，完善市县乡村四级公共文化网络。加强公益文化场馆建设，年内启动建设霞光大剧院和丝弦大戏院，打造文化设施新亮点。加强非物质文化遗产保护和利用。支持文化精品创作，打造文化品牌。大力发展文化产业，围绕现代传媒、印刷出版、动漫游戏、民俗文化等六大产业板块，改造传统产业，培育壮大新兴业态，做大做强报业、广电、演艺三大文化企业集团，着力培育一批主业突出、竞争力强的大型文化企业。年内启动建设动漫大厦和园博园艺术长廊，突出抓好北方书城二期建设，打造华北一流的图书批发交易市场。积极发展卫生事业。加快建立覆盖城乡的居民公共卫生和基本医疗服务体系，加强县乡村医疗机构建设，抓好基层卫生人才队伍特别是全科医生培养，提高基本公共卫生服务均等化和管理服务水平。切实做好公共卫生保障工作，实施“健康知识普及行动”，有效防控重大疾病和传染病。推进市级优质卫生资源倍增工程，市中心医院、市一院、市儿童医院、市四院新院址项目开工建设。维护社会和谐稳定。加大社会管理创新推进力度，在110综合警务站服务功能完善、流动人口一证通、社区建设、物业管理、创新互联网管理模式等方面实现新突破。加强信访和行政复议，畅通群众利益诉求渠道，维护公平正义。加强重点行业和领域安全生产监管，严格落实安全生产责任制，强化安全防控措施，坚决遏制重特大事故发生。健全公共安全应急机制，提高突发事件应急处置能力。进一步完善社会治安防控体系，严厉打击各类刑事犯罪，深入开展“打黑除恶”专项行动，始终保持对犯罪分子的高压态势，切实保障人民群众生命财产安全。加快发展体育等各项事业。广泛开展全民健身运动，不断提高竞技体育水平，开工建设全民健身（游跳）中心，沿环城水系基本建成宽3米、长100公里的环城自行车健身绿道。继续做好人口和计划生育工作，综合治理出生人口性别比偏高问题，稳定低生育水平。依法保障妇女和未成年权益，关心支持残疾人事业。开展“六五”普法，加强法制宣传教育。全面贯彻党的民族政策，依法管理宗教事务。支持工会、共青团、妇联等人民团体发挥桥梁纽带作用，做好外事侨务、防震减灾、气象人防、社科档案等各项工作。加强国防教育和国防后备力量建设，深入开展双拥共建，巩固发展军政军民团结。

（九）实施民生幸福工程，持续提升人民群众的幸福感。今年市级财政安排20亿元集中实施十大民生工程：一是实施就业保障工程。进一步完善培训提高、就业帮扶、创业带动三位一体的就业服务体系，强化对高校毕业生、退役军人、农村转移劳动力、城镇就业困难人员等重点人群的就业服务，着力提升就业技能和就业质量，确保城镇新增就业9.9万人，实现农村劳动力转移15万人，全市城镇登记失业率达到省控指标以下,保持零就业家庭动态为零，应届高校毕业生登记失业率为零。扩大保险覆盖面，城乡居民养老保险制度实现全覆盖。继续提高企业退休人员基本养老金和城乡低保标准，确保高于全省平均水平。推进医疗保险市级统筹，提高基本医疗保障水平。加快建立政府主导和社会参与、居家养老和机构养老相结合的养老服务体系，重点推进敬老院、光荣院、福利院“三院合一”建设，年内示范性老年公寓建成投用，新建农村互助幸福院1100个。关心城镇特困群体，完善社会救助体系，健全保障标准与物价上涨挂钩联动机制，及时发放临时价格补贴。二是实施教育扶贫工程。加大市县两级财政投入力度，年内再新改建28所寄宿制中小学，确保深山区中小学生全部实现集中就读。把发展职业教育作为教育扶贫工程的关键一环，大力改造提升县级职业教育水平，使每一个未能考上大学的贫困山区孩子都能接受良好的职业教育，掌握一技之长，以此改变自己和家庭的命运，从根本上实现脱贫。三是实施医疗惠民工程。进一步完善基层医疗卫生服务网络，健全城市社区医疗保障网络，抓好乡镇卫生院农村卫生室一体化管理，全面提升农村基层医疗卫生机构管理服务水平，做到农民群众小病不出村，常见病和多发病不出乡，大病不出县。新增60所政府办社区卫生服务机构，市区每个街道办事处设置1所政府办社区卫生服务中心，做到城市居民大病到医院，小病康复在社区。继续落实基本药物制度，50%的县级医院实行基本药物零差率销售，新农合筹资标准由230元提高到290元，最高支付额由6万元提高到8万元，切实解决群众“看病难、看病贵”问题。四是实施食品安全工程。以创建食品安全最放心城市为目标，强化监管手段，实行综合执法，提升食品安全监管效能。深入开展“食品安全提升年”活动，加大食品安全专项整治力度，建立肉类蔬菜流通追溯体系，对重点区域、重点领域、重点企业、重点环节、重点品种实施更加科学严格的监管，确保群众饮食放心。今年市内五区各规范建设10个餐饮服务食品安全示范店。五是实施住房保障工程。继续加大廉租住房和公共租赁住房建设力度，在保证工程质量的前提下，加快施工进度，确保按时保质完成任务。全年开工建设各类保障性住房3.69万套，确保5000套租赁住房分配到保障家庭，6300万元补贴发放到2万户廉租住房保障家庭。六是实施蓝天碧水工程。突出抓好城市大气、水污染控制和治理，以国家提高对省会城市大气环境质量考核标准为契机，综合整治全市394家建材企业，治理西柏坡高速两侧可视范围内82家建材和小水泥企业，主城区范围内623台分散燃煤锅炉两年内全部拆除或改电改气，大力发展天然气、沼气、太阳能清洁能源，提高清洁能源使用率。加强水源地水质保护，确保岗南、黄壁庄水库主要水质指标稳定保持在国家地表水二类水质标准，主要河流监测断面水质达到省控标准。开展大规模植树绿化活动，重点抓好西柏坡高速、三环公路、京石铁路、京珠高速、307和308国道市区段、主城区到四组团道路两侧的植树绿化，推进西部山前、环城水系和滹沱河两岸绿色长廊工程建设，今年完成造林绿化17万亩，全市森林覆盖率达到30.6%。七是实施便民市场工程。进一步完善市场建设规划，抓好各类市场的建设、改造、提升。积极发展便利店、肉菜店、餐饮店、洗衣店以及修理维护等便民利民的生活服务业项目，方便市民日常生活。大力推进早餐工程和放心馒头工程建设，积极扶持龙头企业发展，规范馒头等主食品市场和早餐车经营管理，方便人民群众生活。年内建成60家具有网上开店、科技服务、质量控制等多功能的放心农资超市。八是实施交通改善工程。大力提升主城区路网承载能力，完成友谊大街改造等8条续建道路，打通建明中路等5条断头路，整修市区破损严重的小街巷30条。全方位加强城市交通管理，改善交通秩序，充分利用现有道路资源，最大限度提高城市道路通行能力。新开辟5条城乡公交线路，购置新型公交车450辆。实施农村公路“村村连通”建设，年内完成农村公路120公里。九是实施供热提质工程。全面加强市区热源热网建设，确保小区入住与供热保持同步。大力提高老旧小区供热质量，年内完成120个老旧小区供热二次管网改造，确保采暖期不能让一户老百姓受冻。十是实施文化惠民工程。继续办好燕赵讲坛、高雅艺术演出、群众性书画展等形式多样、丰富多彩的文化活动，在城乡免费放映电影5万场以上，每村每月放映一场，“彩色周末”群众性文艺演出1000场以上，专业艺术院团城乡演出800场以上。市图书馆、群艺馆、博物馆、美术馆、民间工艺博物馆等公益场馆实行365天免费开放。

各位代表！保障和改善民生是我们义不容辞的责任，在此，我代表市人民政府向全市人民郑重承诺：我们将努力做到像抓经济建设一样抓民生保障，像落实经济指标一样落实民生工程，加快推进速度，加大落实力度，确保如期圆满完成。

四、切实加强政府自身建设

新形势新任务对政府工作提出了更高要求，新目标新举措要求我们必须以改革创新精神，切实加强政府自身建设，加快思想转型，推进能力升级，全面提升推动科学发展的执行力，切实肩负起“转型升级、跨越赶超”的历史重任。

（一）奋发有为，保持良好精神状态。面对竞相发展的激烈竞争，市政府各部门必须切实增强责任感、紧迫感、使命感，强化机遇意识、忧患意识、进取意识，大力弘扬不甘落后、奋勇争先的精神，面向全国找准坐标点，对标先进找准起跑线，努力实现争先进位。必须大力弘扬勇于担当、敢为人先的精神，坚决破除陈旧观念和惯性思维，突破制约发展的体制机制障碍，推动各项工作上水平。必须大力弘扬求真务实、真抓实干的精神，始终保持一个好的精神状态、一个好的工作作风、创造一流的工作业绩，以旺盛的工作热情和强烈的事业心，不断推动全市经济社会发展迈上新台阶。

（二）善于学习，提高科学发展能力。坚持把学习作为一种政治责任和精神追求，深入系统地学习政治理论和现代知识，不断更新知识结构，树立开放的思想观念，打开解放思想的总开关，用改革的办法、创新的思维解决新矛盾新问题。系统研判宏观经济形势和产业发展趋势，深入了解国家产业、土地和金融政策，掌握经营管理城市和经营管理企业的基本常识，以自身知识结构的转变、能力素质的提高，推动全市产业加快升级、发展方式加快转变。坚持把中央和省委、省政府的决策部署与石家庄市情相结合，尊重科学，遵循规律，实事求是，创新工作思路，创新方式方法，唱好地方戏，打好特色牌，营造新亮点，努力在改革创新中开拓省会发展进步新天地。

（三）高效服务，一心为民谋福祉。牢固树立以人为本、执政为民的理念，带着感情、责任和追求，实实在在地为百姓解难事、办实事、做好事。坚持把优化发展环境作为一项“永不竣工的工程”，全面推行公开承诺制、首办责任制、限时办结制、考核评议制，提速办事效率，提高服务质量。大力精简审批事项，该取消的坚决不能保留，该下放的坚决予以下放，努力创造在全省审批项目最少、程序最简、费用最低、服务最优的服务环境。

（四）强化责任，脚踏实地抓落实。围绕各项目标任务，逐级建立主体明确、责权对称、层级清晰、奖罚分明的责任体系。以责任制促落实，以责任制保成效，形成一级抓一级，层层抓落实的工作局面。切实加强对重大决策落实情况的监督检查，确保政令畅通，加强绩效管理，落实行政问责，实行责任追究。进一步改进文风会风，开短会、讲短话、求实效，排除一切干扰，摒弃私心杂念，把心思和精力集中到干事创业上，以“踏石有痕、抓铁留印”的过硬作风，扎实推进各项工作落到实处。

（五）依法行政，真心实意受监督。忠实履行法律赋予政府的各项职责，把依法行政贯穿到政府工作的各个领域、各个环节，严格按照法定权限和程序想问题、做决策、办事情。认真接受人大的法律监督、政协的民主监督和社会舆论监督，广泛听取各民主党派、工商联和无党派人士的意见和建议。建立健全公众参与、专家论证和政府决定相结合的行政决策体制，真正做到科学决策、民主决策。进一步健全行政执法体制规范管理模式，完善运行机制，激励行政执法人员严格执法、公正执法、文明执法，让公平正义的阳光普照到每一位公民。

（六）廉洁从政，树立政府好形象。全面贯彻“标本兼治、综合治理、惩防并举、注重预防”的方针，进一步加强廉政勤政教育，切实提高公务员队伍廉洁从政的自觉性。坚持以制度建设为主线，以权力监控为核心，建立健全惩治和预防腐败体系，从源头上防止和减少腐败问题的发生。深入开展民主评议活动，坚决纠正行业不正之风，切实维护人民群众的合法权益，始终做到勤政廉政、干净干事，确保我们所做的各项工作经得起历史的检验，对得起养育我们的人民，努力建设一个人民满意的政府。

各位代表！做好今年的工作，任务艰巨，责任重大，使命崇高。让我们在省委、省政府和中共石家庄市委的坚强领导下，深入贯彻科学发展观，认真落实中央和省的一系列决策部署，坚定信心，迎难而上，开拓进取，扎实工作，奋力开启建设幸福石家庄的新征程，谱写石家庄又好又快发展的新篇章，以科学发展的新成绩迎接党的十八大胜利召开！
